# 

**Published:** 1847-10

**Authors:** 


					y f
THE
BRITISH AND FOREIGN .. $
. ' ;.0<v>*V,y
3i ^
:Ig gournal
M.D. F.II.S. F.G.S.
OE OF PHYSICIANS IN tONDON.
-ott'^f^i
B BBITISM AND FOBEIGN MED*
?iblisied, oo tkeJst of January,
UNDER THE TITLE OF THE
v ,r V : 7: >
MEDICO-CHIBURGICAL
. xp*?sa*^sm*
REVIEW,
(?uartcrtg journal of practical
,
mm I'Mm
m
*** ?*&&.. 2* it ~ <
ILL AND SAMUEL HIGHL1
OjDOMEW CLOSE. | (J
PRICK SIX SHILLINGS.

				

